# Exploring the Impact of Time Spent Reading Product Information on E-Commerce Websites: A Machine Learning Approach to Analyze Consumer Behavior

**DOI:** 10.3390/bs13060439

**Published:** 2023-05-23

**Authors:** Sabina-Cristiana Necula

**Affiliations:** Department of Accounting, Business Information Systems and Statistics, Faculty of Economics and Business Administration, Alexandru Ioan Cuza University of Iasi, 700505 Iasi, Romania; sabina.necula@uaic.ro

**Keywords:** e-commerce, clickstream data, Machine Learning, Principal Component Analysis, clustering

## Abstract

In this study, we aim to investigate the influence of the time spent reading product information on consumer behavior in e-commerce. Given the rapid growth of e-commerce and the increasing importance of understanding online consumer behavior, our research focuses on gaining a deeper understanding of customer navigation on e-commerce websites and its effects on purchasing decisions. Recognizing the multidimensional and dynamic nature of consumer behavior, we utilize machine learning techniques, which offer the capacity to handle complex data structures and reveal hidden patterns within the data, thereby augmenting our comprehension of underlying consumer behavior mechanisms. By analyzing clickstream data using Machine Learning (ML) algorithms, we provide new insights into the internal structure of customer clusters and propose a methodology for analyzing non-linear relationships in datasets. Our results reveal that the time spent reading product-related information, combined with other factors such as bounce rates, exit rates, and customer type, significantly influences a customer’s purchasing decision. This study contributes to the existing literature on e-commerce research and offers practical implications for e-commerce website design and marketing strategies.

## 1. Introduction

The burgeoning e-commerce landscape has positioned the study of consumer behavior in online transactions at the forefront of critical research. Prior investigations have spotlighted elements such as technology [1,2,3,4,5,6], trust [7,8,9], and convenience [10,11,12] as pivotal determinants of consumer behavior. However, the full picture remains elusive. Key insights have demonstrated the significant influence of product delivery and associated costs on consumer decision making [13]. Furthermore, these factors have been identified as crucial drivers of the online shopping market during the COVID-19 pandemic [14], paving the way for new research opportunities.

An underexplored aspect of this domain is the impact of the time consumers invest in reviewing product information on e-commerce websites. This research gap is accentuated by the complexities associated with gleaning user activity features from intricate data [4].

Survey data from 2020 [15] suggested an anticipated uptick in online purchases in the post-COVID-19 era compared to pre-pandemic times. However, 2022 studies revealed that this surge in online expenditure was more prevalent in economies where e-commerce was already a significant player. Interestingly, this trend seems to be reversing as the pandemic wanes [16]. Nevertheless, the global e-commerce landscape is replete with promising trends fueled by a host of factors, notably technological advancements [17].

Machine learning (ML) is progressively adopted in marketing research, enabling the development of predictive models and the discovery of non-linear relationships in data. Existing studies have leveraged ML to pinpoint high-risk customers for retention programs [18], predict and understand the causes of customer churn through classification algorithms such as Random Forest [19], and examine customer consumption behavior using improved radial basis function classifiers and oversampling methods [20]. Deep learning models such as neural networks and deep Markov models have been employed to forecast online shopping behavior. Research indicates that a blend of RNN and traditional classifiers outperforms other models [21]. Meanwhile, attentive deep Markov models have been used to predict the risk of user exits without purchase during e-commerce sessions [22]. Additionally, methods such as the L1-and-L2-norm-oriented Latent Factor Model have demonstrated significant potential in recommender systems [23]. These advancements provide valuable tools for predicting customer behavior and understanding churn risks.

Against this backdrop, our study’s primary goal is to examine the influence of time spent reading product information on consumer behavior in e-commerce. Through the analysis of clickstream data, we aim to delve deeper into the patterns of customer navigation on e-commerce websites and its impact on their purchasing decisions.

### Motivation

As e-commerce continues its rapid expansion, gaining insights into how consumers interact with and navigate online shopping platforms becomes crucial for businesses. Notably, the time consumers allocate to reading product information presents a substantial yet unexplored facet of online consumer behavior. Investigating this area could shed light on consumer decision-making processes, potentially informing more effective website design and marketing strategies [4]. Moreover, research on user retention mechanisms, such as in online learning platforms, adds a new dimension to the understanding of continuance intention behavior [24].

Our study offers two main contributions to the field of e-commerce research. Firstly, it enhances our understanding of the internal structure of customer clusters and its correlation to consumer behavior. Secondly, it introduces a methodology for analyzing datasets with non-linear relationships using ML algorithms [23].

The structure of this article is divided into three primary sections. Section 3; outlines the research design and methodologies employed in the study. Section 4 presents the study’s findings. Finally, Section 5 offers a detailed analysis of the results and their implications for practice and future research.

In conclusion, our study addresses a crucial gap in the understanding of e-commerce consumer behavior, focusing on the influence of time spent reviewing product information on e-commerce websites. We provide fresh insights into the internal structure of customer clusters, the mechanics of user retention [24], and a methodology for analyzing non-linear relationships [23]. By doing so, our research enriches the existing body of knowledge in the e-commerce domain, offering valuable pointers for effective website design, marketing strategies, and potential avenues for future investigation.

## 2. Literature Review

Scientific literature on e-commerce is predominantly empirical, focusing on validation of theoretical constructs and hypothesis testing. A critical tool in this field, ML, has been extensively used for diverse purposes such as customer behavior analysis, recommender system development, and predictive model construction [12,25]. ML models have demonstrated proficiency in predicting customer purchase intentions and identifying additional products likely to interest users.

Current e-commerce research leverages advanced technologies such as augmented reality [26,27], electronic word-of-mouth (eWOM), review analysis, social media [28,29,30,31], haptic sensations, and voice shopping [3,32,33]. These technologies have been harnessed to enhance user engagement and personalize the shopping experience. ML plays a pivotal role within these technologies, as exemplified by recent innovative applications. Chen et al. applied ML in analyzing the impact of population gender ratio imbalance on FinTech innovation [12], while Li and Sun used an ML-based support vector machine for intelligent investment strategies in the stock market [25].

Our study, however, adopts a unique approach by concentrating on the duration customers spend on product-related and administrative pages. We propose that this angle offers invaluable insights into customer decision making, a dimension often overlooked in the current literature [34,35,36].

While clickstream data hold promise for understanding customer behavior in e-commerce, studies on this topic are relatively sparse due to data availability limitations and privacy concerns. Future research is anticipated to concentrate on extracting user behavior information, such as mouse movement and clicks, session times, searched items, and product customization [37]. Studies on real-time customer purchase prediction based on navigation data [38,39,40,41] have yielded promising results using various ML algorithms. Other studies have employed different methods such as Markov chains [42] to identify at-risk users, and gradient boosting with regularization [43] to predict online shopping cart abandonments. These studies aimed to develop accurate ML models for user behavior prediction. Furthermore, research by [44] explored detecting high-purchase-intent users upon their connection to the e-commerce website and offering personalized content.

Recent advancements in deep learning, such as the hyperparameter learning framework by Wu et al. (2023) [36], bear promising implications for recommender systems performance improvement in e-commerce. Likewise, the work of Lu et al. [34] on multiscale feature extraction and fusion in Visual Question Answering (VQA) systems could offer innovative ways to enhance e-commerce platform user experiences.

Whereas previous studies focused primarily on specific web pages that influence a customer’s decision to complete a purchase or leave the website, our method examines the influence of additional factors derived from clickstream data. This holistic approach allows us to capture nuanced behaviors of online shoppers, often overlooked by traditional methods. Contrary to existing methods that rely on simple metrics such as page views or click-through rates, our method employs machine learning to analyze intricate patterns in clickstream data, offering a more accurate and comprehensive understanding of customer behavior, leading to more effective strategies for customer retention and conversion.

## 3. Materials and Methods

The aim of our research was to determine the factors that influence a customer’s decision to make a purchase by analyzing e-commerce clickstream data. The dataset we used is publicly available [38,45] and contains information about user sessions on an e-commerce website, including time spent on product pages, administrative pages, exit rates, bounce rates, and visitor type. As exit rates, bounce rates, and time spent on product information are established indicators of purchase intent, our goal was to identify the best predictors for building ML models. Ultimately, we sought to gain insight into the quality and quantity of product information and delivery options provided on the e-commerce website.

To achieve this goal, we built ML classifiers and evaluated their performance as a validation technique. The target variable in our analysis was “Revenue,” which was represented as a binary variable: 0 for sessions that did not result in a purchase and 1 for sessions that did. The dataset included 12,330 sessions spanning a 1-year period and represented a diverse range of users to avoid any bias towards specific campaigns, days, or user profiles. The dataset was imbalanced, with 84.5% (10,422) being negative class samples and the rest (1908) being positive class samples. It included 10 numerical and 7 categorical attributes as independent variables.

We first conducted descriptive statistics to explore the variables in the dataset. However, as the dataset included both numerical and categorical variables, we extended our analysis to provide a more comprehensive understanding.

The variables Administrative, Informational, ProductRelated, Administrative_Duration, Informational_Duration, ProductRelated_Duration, and Product_Related represent the number of different types of pages (administrative, informational, and product-related) visited by the customer in each session, as well as the total time spent on these pages. BounceRates represents the percentage of visitors who enter the site from that page and then leave, while ExitRates represents the percentage that were the last in the session. PageValues is the average value for a web page that a user visited before completing an e-commerce transaction. The dataset also includes information about the operating system, browser, region, traffic type, visitor type (returning or new), a Boolean value indicating whether the date of the visit is on a weekend, the month of the year, and if the visiting moment is close to a special day.

The research methodology for this study involved the following steps:

1. Formulating the research hypothesis
2. Conducting exploratory data analysis
3. Building ML models
4. Finding the optimal parameters for each model and the best performance metrics
5. Identifying the most important predictors
6. Validating the research hypothesis.

The research hypotheses were:

1. The characteristics of e-commerce website visitors are varied and are influenced by a combination of variables such as the time spent on reading product-related information, the time spent on administrative pages, bounce rates, exit rates, and customer type (returning or new).
2. The proportion of variables related to product and delivery/administration information is significant in influencing a potential customer’s purchasing decision.

The methods used in this study were:

1. For exploratory data analysis, we employed descriptive statistics, box plots, pair plots, and clustering techniques. We also evaluated the validity of clusters using specific metrics, as discussed in the article.
2. We constructed logistic regression, decision tree, random forest, and support vector machine classifiers and validated the models using the Stratified KFold validation technique. We found the optimal parameters using the GridSearch technique.
3. We used metrics such as accuracy, confusion matrix, and ROC_AUC to evaluate the models.
4. We used Python and several libraries including scikit-learn, statsmodels, pandas, and numpy to perform data analysis and model building.

The approach taken in this research included conducting Exploratory Data Analysis (EDA) on a dataset of 12,330 records of customer interactions with an e-commerce website. The goal was to identify factors that contribute to a customer’s decision to make a purchase. To achieve this, we grouped the customer sessions in order to uncover common characteristics among them. As the dependent variable in the dataset was binary, we used ML classifiers such as Logistic Regression, Decision Tree, Random Forest, and Support Vector Machines to validate our assumptions and build models that can be applied to the entire dataset.

### 3.1. Novelty and Significance of the Proposed Method

In this study, we propose a novel approach to understanding customer behavior in the e-commerce field by utilizing clickstream data in combination with Machine Learning (ML) methods. While existing studies have focused on analyzing specific web pages that influence a customer’s purchasing decision, our method considers a broader range of variables, including the time customers spend on pages related to product information and administrative issues.

This represents a significant step forward in the field as it allows for a more comprehensive understanding of customer behavior, going beyond the typical focus on product pages. Our approach can help e-commerce businesses to enhance their website design and content, ultimately improving customer engagement and conversion rates.

### 3.2. Detailed Application of Machine Learning in Analyzing Consumer Behavior

To analyze consumer behavior, we applied several machine learning methods to the clickstream data, including Logistic Regression, Decision Tree, Random Forest, and Support Vector Machines. However, we did not apply these methods directly; instead, we modified them to specifically cater to the nature of our data and the objectives of our study.

We employed GridSearchCV for parameter tuning to find the optimal parameters for each ML model, which is a significant modification from traditional direct application. We also used the StandardScaler to scale the numerical variables, a necessary step given the stochastic nature of the gradient solvers used in our models.

### 3.3. Workflow and Time Complexity Analyses of the Proposed Method

To clarify the process involved in our proposed method, we provide the following pseudocode (Table 1):

The time complexity of our proposed method largely depends on the machine learning models used and the size of the dataset. For instance, the time complexity of the Logistic Regression and Support Vector Machines algorithms is typically O(n^3^) and O(n^2^) respectively, where n is the number of features. However, the GridSearchCV process we used for parameter tuning adds an additional layer of complexity as it involves training and validating the models multiple times for each combination of parameters. Despite this, our method remains computationally feasible given the size of our dataset.

We did not directly apply the existing machine learning methods. Instead, we adapted them to our data and the objectives of our study through preprocessing and hyperparameter tuning. This allowed us to create models that accurately predict customer purchasing decisions based on e-commerce clickstream data.

### 3.4. Building and Validating Machine Learning Models

Machine Learning (ML), a subset of Artificial Intelligence (AI), has been broadly used in e-commerce research for prediction, classification, and clustering. Existing ML models, including Logistic Regression, Decision Tree, Random Forest, and Support Vector Machines, have shown promise in analyzing customer behavior data. Our proposed method, however, introduces a novel approach to ML application in e-commerce, focusing on clickstream data analysis.

Our ML models build on existing models’ strengths while addressing some of their limitations. For instance, while Logistic Regression and Decision Tree are known for interpretability, they may not be as accurate when dealing with complex, non-linear relationships in the data. Conversely, while Random Forest and Support Vector Machines can capture these complex relationships, they may be prone to overfitting and lack interpretability. Our proposed models are designed to balance accuracy and interpretability, rendering them more suitable for analyzing clickstream data. Furthermore, our models are modified to tackle the unique challenges of clickstream data, such as high dimensionality and temporal dependencies.

Logistic Regression, a linear model of regression utilizing L2 regularization (also known as Ridge Regression or L2 norm), is a commonly used ML algorithm in statistics and data analysis. It is typically employed to separate data points with a linear regression line. However, compared to other non-linear ML models, it may fall short in terms of accuracy. For logistic regression, we used the logistic regression class from the scikit-learn library in addition to the ordinary least squares method from the statsmodels package. Instead of directly applying the logistic regression, we searched for the best values of the hyperparameters by using GridSearchCV (Grid Search Cross Validation). We found that the ‘saga’ solver improved logistic regression performance over the default ‘lbfgs’ solver. Saga is an optimization algorithm extension of Stochastic Average Gradient (sag) for calculating loss, which also allows for L1 regularization. It generally trains faster than sag and tends to converge faster if data is not on the same scale.

The Decision Tree ML algorithm, part of the Classification and Regression Tree Algorithms (CART) family, has been employed widely in both scientific literature and practice. The algorithm splits each variable’s values and builds a tree. Despite being a fast algorithm, it tends to overfit data [37]. For the decision tree algorithm, we used the DecisionTreeClassifier class from the scikit-learn library. However, instead of directly applying it, we conducted a grid search to find the optimal value for its key parameter, max_depth. By tuning this parameter, we could control the depth of the tree to prevent overfitting.

Random Forest, an ML algorithm, constructs multiple decision trees and averages the decisions of multiple trees trained on different portions of the same training set, aiming to reduce the unit standard deviation. For the random forest algorithm, we used the RandomForestClassifier from the scikit-learn library. We conducted a grid search to determine the optimal values for the number of features (no_of_features) to use and the maximum depth of the trees (max_depth). Tuning these parameters allows us to build an ensemble of decision trees that perform well on our dataset.

Support Vector Machines (SVM) separates data points using a set of hyperplanes in a multi-dimensional space. The larger the hyperplane margin, the lower the classifier’s generalization error [46]. Besides linear classification, SVMs can perform non-linear classifications using the kernel trick, involving different kernel functions. For the support vector machine (SVM) algorithm, we used the SVC class from the scikit-learn library. The standard SVM was modified by using different kernel functions to transform the data, with the best known being radial basis kernel function (‘rbf’ in scikit-learn).

In all cases, we preprocessed our data by scaling the numerical variables using the StandardScaler from the scikit-learn library. This step is crucial due to the stochastic nature of the gradient solvers used in our models, which require features to be on the same scale for optimal performance.

The performance of each ML algorithm was compared using various metrics, including AUC (Area Under the Curve) ROC (Receiver Operating Characteristics), confusion matrix, F1 score, and accuracy. An ideal model exhibits an AUC close to 1, indicating good separability. These metrics all leverage true positives, false positives, true negatives, and false negatives. Performance calculation formulas include accuracy, recall or sensitivity, specificity, and the ROC curve, which plots the recall (true positive rate) versus false positive rate. The area under the ROC curve approximates good output values, and its value should approach 1 to evaluate a binary classifier’s performance as excellent.

Clustering, an unsupervised ML method, is used to identify related record groups. The KMeans algorithm, which uses Euclidean distance to measure the distance between data points and assign them to clusters, is the primary algorithm employed in clustering. However, the requirement to specify the number of clusters in advance is a key disadvantage of this algorithm. This study used the silhouette, Calinski-Harabadz, and Davies-Bouldin indexes to find the optimal number of clusters and validate the final clustering.

The KMeans algorithm may not perform well on datasets with multiple features. Therefore, a dimensionality reduction technique, such as Principal Component Analysis, should be applied in advance. This study used the Principal Component Analysis technique and examined the cumulative explained variance to compute the optimal number of features required for training the KMeans algorithm.

Principal Component Analysis (PCA) [47] is a powerful statistical technique used for various purposes, including revealing relationships between variables and relationships between samples (e.g., clustering), detecting outliers, and generating new hypotheses. PCA works by transforming the original variables into a new set of variables, the principal components, which are uncorrelated and account for the variance in the data in decreasing order. The first few components often capture a substantial proportion of the data’s variability, allowing for a reduction in dimensionality without significant loss of information.

The first step of PCA is to compute the covariance matrix of the data, which measures how much each pair of variables varies together. The eigenvectors and eigenvalues of the covariance matrix are then calculated. Each eigenvector corresponds to a principal component, and the corresponding eigenvalue indicates the variance explained by that component. The eigenvectors are typically ordered by decreasing eigenvalues, so the first principal component is the direction in the data space along which the data vary the most.

PCA has been used extensively in e-commerce research for analyzing high-dimensional customer data. The application of PCA to clickstream data enables the extraction of the most important features that influence customer behavior, which can improve the accuracy of ML models. Our proposed method uses PCA to reduce the dimensionality of clickstream data before applying the KMeans algorithm, which can improve the clustering performance.

Despite the potential of PCA and KMeans for analyzing customer behavior in e-commerce, there are relatively few studies on the topic due to the complexity of the methods and the need for high-quality data. Our research aims to address this gap by demonstrating how PCA and KMeans can be effectively applied to clickstream data to extract valuable insights about customer behavior. By exploring the time spent by customers on different web pages and their navigation patterns, we aim to provide a more nuanced understanding of customer behavior, which can inform the design of more effective e-commerce strategies.

To validate the performance of the PCA and KMeans methods, we use several metrics, including the silhouette score, the Calinski–Harabadz Index, and the Davies–Bouldin Index. By comparing the performance of different models and configurations, we aim to identify the most effective methods for analyzing clickstream data. We also explore how different preprocessing steps, such as feature scaling and outlier detection, can impact the performance of the PCA and KMeans methods.

We believe that our research can provide valuable insights for e-commerce practitioners and researchers, and we hope that it will stimulate further research on this important topic.

## 4. Results

This section presents the results of applying the algorithms and techniques discussed in the Section 3.

### 4.1. Exploratory Data Analysis

Table 2 contains the descriptive statistics for all the variables. The mean, standard deviation, maximum, minimum, and quantiles values indicated a right skew of all variables. However, since the dataset consists of data stored in numerical and categorical variables, the analysis was extended to provide better insights.

In order to gain a more comprehensive understanding of the dataset, we chose to analyze outliers as part of our analysis strategy. This helped us to identify any patterns or trends that may have been obscured by the presence of extreme values in the data.

**Figure 1:**
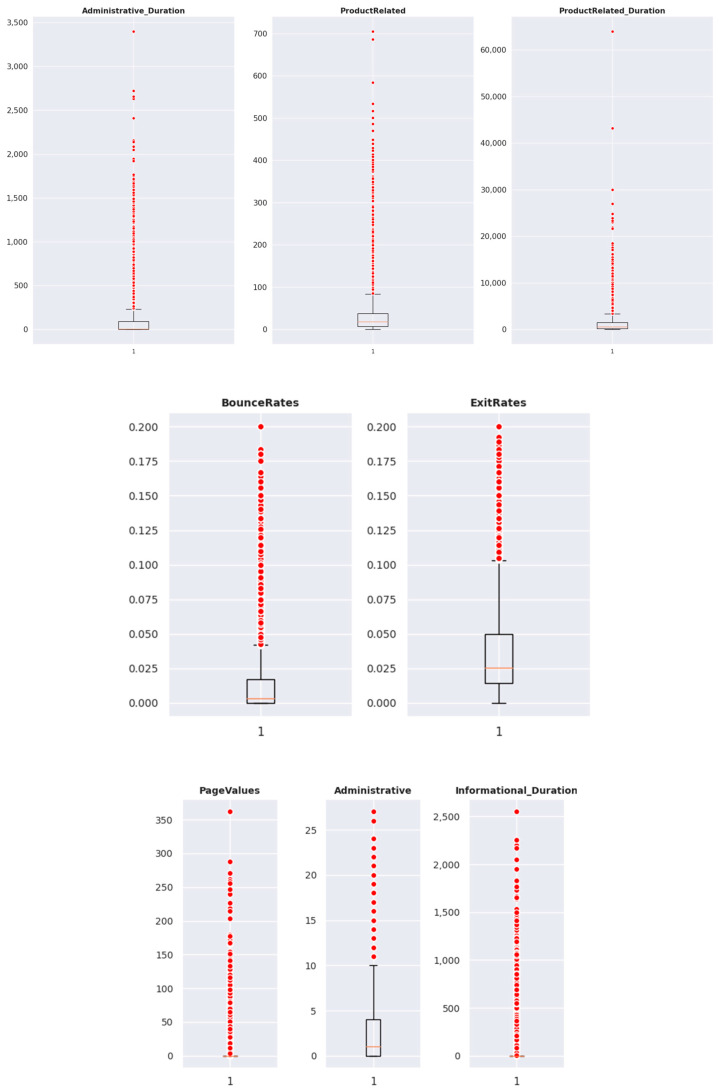
The box plots of numerical variables.

Figure 1 illustrates the box plots of numerical variables. The box plot is a useful tool in univariate analysis as it visually displays the values of each variable, including median values, outliers, lower range, and upper range.

**Figure A1:**
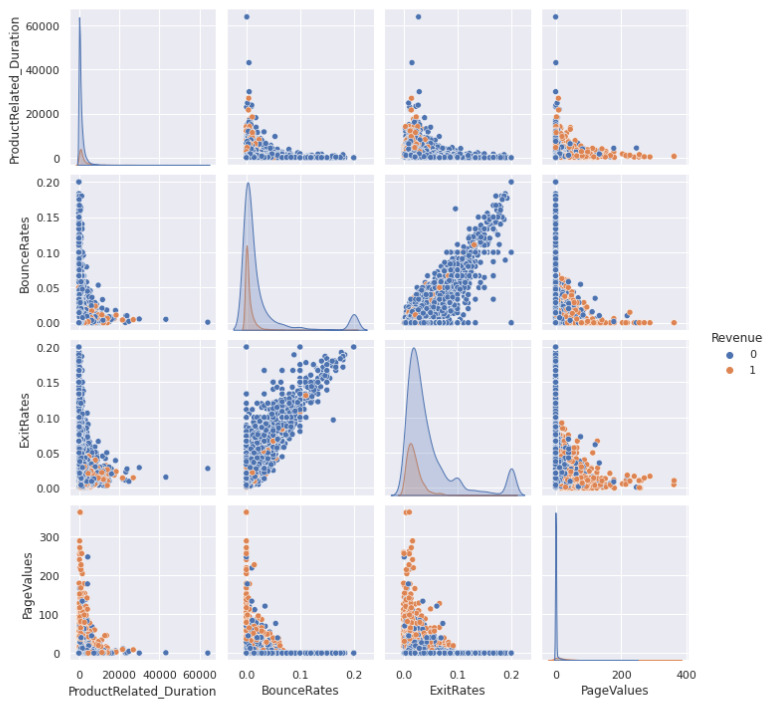

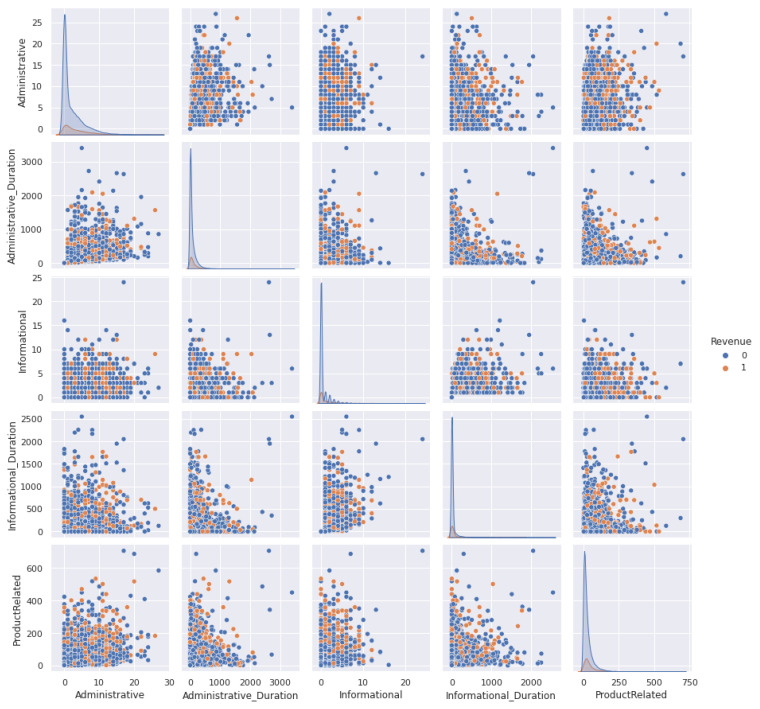
The pair plots of numerical variables.

**Figure A2:**
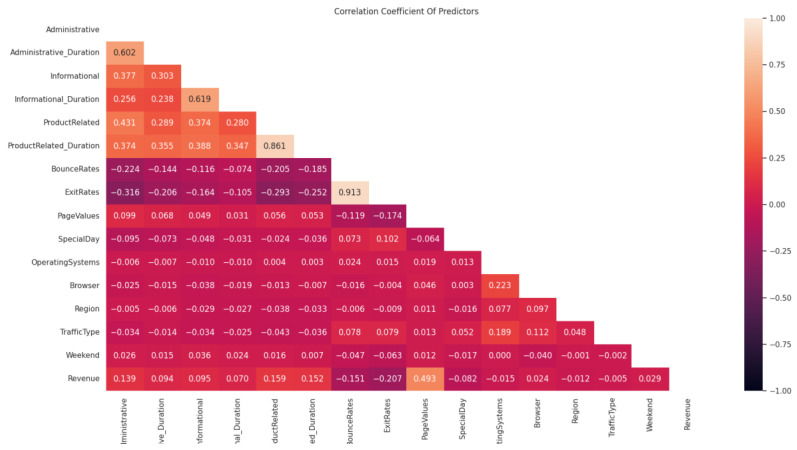
Heatmap.

**Figure A3:**
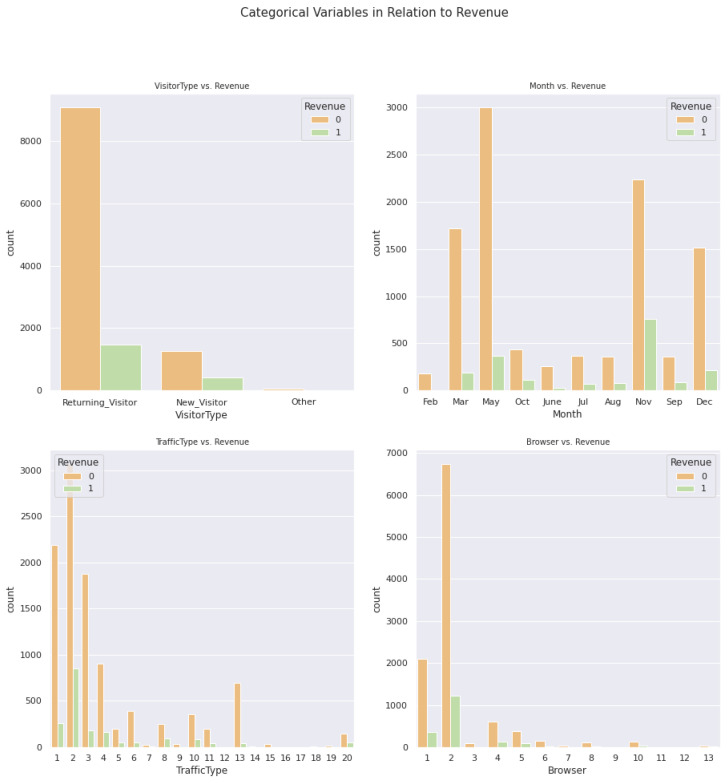
The count plots for categorical variables.

As shown in Figure 1 and Table 2, there are a significant number of outliers present in the dataset. The minimum value for each variable is positive, and the median value is less than the mean value for most variables. This suggests that different ML models can be built to predict the “Revenue” outcome. However, we also examined the distribution plots and the correlation matrix to gain further insights. Figure A1 in the appendix illustrates the pairwise correlations between variables, and Figure A2 presents a heatmap of these correlations. The Bounce Rates and Exit Rates are highly correlated, as are the ProductRelated and ProductRelated_Duration variables. The categorical variables, as illustrated in Figure A3, show an imbalanced right skewness of frequency values and, as observed in the heatmap of correlations, have no significant influence on any other variable. We conducted various tests and evaluated the values of the outliers and determined that each outlier is important for our analysis.

### 4.2. Cluster Analysis

**Figure 2:**
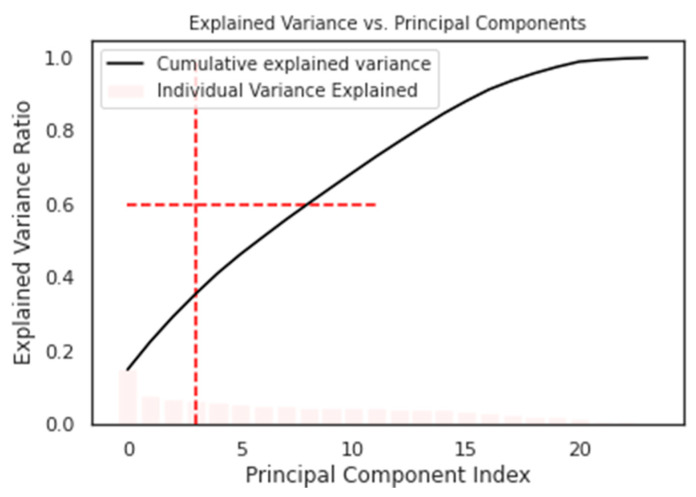
Explained variance versus principal components.

The cluster analysis and Principal Component Analysis (PCA) were employed to identify the optimal number of features to predict the outcome, with nine components determined as the ideal number (as shown in Figure 2).

**Figure 3:**
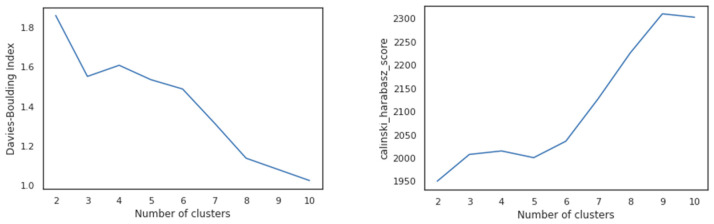

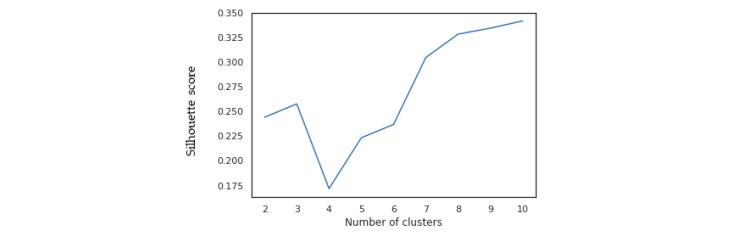
The silhouette, Callinski–Harabadz, and Davies–Boulding Index scores.

We used dimensionality reduction techniques, specifically Principal Component Analysis (PCA), to determine that nine components constituted the optimal number for predicting the output variable. We then evaluated these nine components using the silhouette, Calinski–Harabasz, and Davies–Boulding index scores (as shown in Figure 3) to determine the optimal number of clusters. The scores favored the average similarity measure of each cluster with its most similar cluster and, as the resulting number of clusters was substantial, we decided to proceed with nine clusters.

The average values of each variable per cluster are presented in Table 3.

The cluster analysis was conducted to identify differences between clusters. Upon analyzing the mean values, we found that certain variables did not show significant differences between clusters and thus decided to drop the BounceRates, OperatingSystems, Browser, Region, TrafficType, and Weekend variables from further analysis. Additionally, we found that BounceRates were highly correlated with ExitRates and thus dropped the BounceRates variable to reduce multicollinearity.

We noted that if we established a threshold of 0.5, the cluster indexed 5 had a mean ‘Revenue’ value of 0.95, indicating that this cluster represents customers who completed e-commerce transactions. Characteristics of this cluster include a higher average time spent researching the product, a larger average value of the page that led to the customer’s purchasing decision, and the highest mean value of the ‘Informational’ variable among all clusters. This cluster can be characterized as the most informed about the product.

The cluster indexed with 0 represents customers who did not complete e-commerce transactions and had the lowest mean values of all variables among all clusters. The other clusters fall in between these two extremes, but our focus was on gaining insights into the variables to improve ML models.

### 4.3. Machine Learning Models Building and Validation

The remaining independent variables used in this study were ‘Administrative’, ‘Administrative_Duration’, ‘Informational’, ‘Informational_Duration’, ‘ProductRelated’, ‘ProductRelated_Duration’, ‘ExitRates’, ‘PageValues’, ‘Month’, and ‘VisitorType’. We then proceeded to build ML classifiers. A 25% sample of the dataset (3082 user sessions) was set aside as test data for validation, while 75% (9248 user sessions) was used as training data. As the classes were highly imbalanced, we used the ADASYN algorithm to oversample the training dataset, resulting in a dataset of 16,058 user sessions to model the classifiers with StratifiedKFold validation and finding the best values for each classifier’s hyperparameters. The accuracy (AUC_ROC score) for the different ML classifiers was calculated using cross-validation with StratifiedKFold validation (cv = 10) and is presented in Table 4.

The results presented in Table 3 indicate that the Random Forest classifier is the most effective model for predicting whether a new customer will make a purchase of a specific product. The performance of each algorithm was optimized through the tuning of their hyperparameters, as shown in Table 5. The metric used to evaluate the performance was the ROC_AUC score.

**Figure 4:**
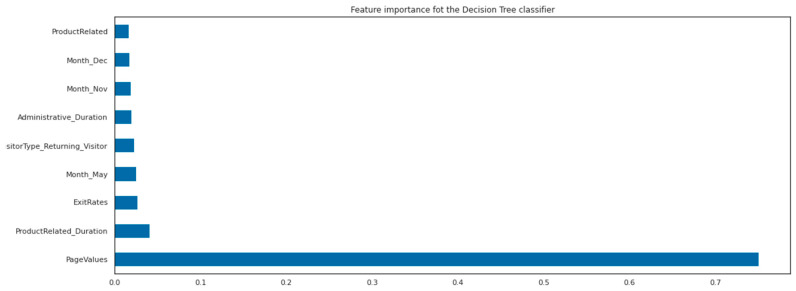
Feature importance of the decision tree classifier.

The results of the GridSearchCV, as presented in Table 5, indicate that the optimal number of features to be used by each tree in the RandomForest classifier is 12, which is less than the total number of features and helps to prevent overfitting. Additionally, the optimal solver and kernel for this dataset were found to be ‘Saga’ and ‘rbf’, respectively, as these are suitable for datasets that are not easily predictable and require the use of non-linear algorithms. The feature importance of the decision tree classifier is illustrated in Figure 4.

**Figure 5:**
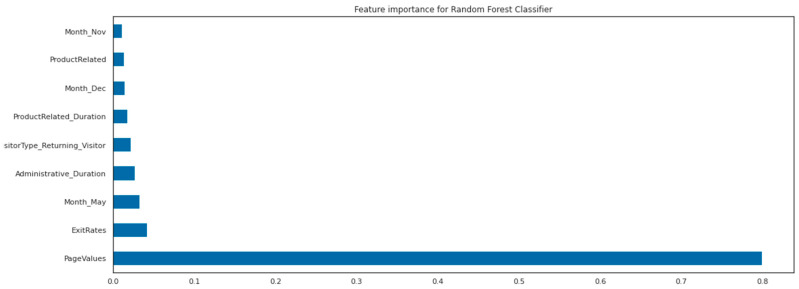
Feature importance of the RandomForest classifier.

In addition to PageValues, ExitRates, whether the customer is a returning visitor, and the month, it appears that the time spent reading ProductRelated information and the time spent on administrative tasks on the website’s pages have a significant impact on the final outcome. These are the results of the decision tree algorithm. The feature importance for the RandomForest classifier is presented in Figure 5.

The results of our analysis indicate that both the Random Forest and Support Vector classifiers performed the best among all the ML models that we constructed. However, after fine-tuning the parameters, we observed comparable performance results across all algorithms. It is noteworthy that the Random Forest classifier identified the same variables as being important in predicting the outcome as the decision tree algorithm. The feature importance for the Random Forest is determined by averaging the feature importance for each individual tree. Notably, the average importance of the time spent on administrative tasks is slightly higher than that of variables related to product information, but both present relatively similar values. We validated our models using a separate portion of the dataset that we extracted before building and validated our ML classifiers with the StratifiedKFold technique. The performance metrics are presented in Table 6.

The results of the training and test data accuracy for all classifiers indicate that the model is neither overfitting nor underfitting, with the exception of the decision tree classifier, which is known for its tendency to quickly learn and overfit. However, the prediction accuracy remains high, with a score above 90% in detecting false negatives. The primary goal of identifying the best predictors is to ensure the classifier can accurately predict the ‘true’ class, as customers who are about to exit the website pages are of particular interest. A useful measure in this case is recall, which aims to predict the ‘1’s’ as accurately as possible. In all cases, the recall is high, indicating that the classifiers are correctly predicting defaulters. Another evaluation criterion of interest is ROC-AUC, which is useful for predicting defaulters. The AUC values are very good, indicating that the classifiers are performing well in predicting both defaulters and non-defaulters.

## 5. Discussion

This study embarked on a comprehensive exploration to understand the key factors that shape consumers’ intent to purchase while navigating e-commerce platforms. Our journey began with an extensive exploratory data analysis of the dataset, which comprised both numerical and categorical variables. Interestingly, the dataset was characterized by a significant number of outliers, which we opted to retain for our analysis. The decision to preserve these outliers was driven by our belief in their potential to enrich our understanding of atypical customer behaviors, which could illuminate unique insights into online purchasing patterns.

Our analysis revealed that the duration of product-related information review and the time devoted to administrative tasks significantly influence customers’ purchase intentions. These findings underscore the necessity of a delicate equilibrium between the provision of relevant, concise product information and the seamless integration of advanced IT technologies to facilitate a satisfactory user experience for e-commerce site visitors. This assertion is in alignment with established research in the field [26,27].

Interestingly, our research indicated that bounce rates did not significantly impact the differentiation of customer clusters. We noted correlations between certain variables and their potential implications on our models. For instance, we found a strong correlation between bounce rates and exit rates. This suggested that bounce rates could be influenced by factors not included in our dataset, such as the visual aesthetics of the website [48] or general trust in e-commerce platforms.

The application of cluster analysis revealed distinct groupings within our data. The focus was directed towards specific clusters that demonstrated unique characteristics pertinent to our research question. These clusters stood out due to their distinct browsing or purchasing behaviors, offering valuable insights into different segments of e-commerce consumers.

In terms of machine learning models, we constructed several but found the Random Forest classifier to be the most effective. Our evaluation was not solely based on the ROC_AUC score but also considered other factors such as interpretability, computational efficiency, and robustness to outliers and noise. Despite the superior performance of the Support Vector Classifier in previous studies, our meticulous data analysis and strategic application of PCA, in combination with clustering, led us to favor the Random Forest model.

Feature importance was visualized and discussed extensively for the Decision Tree and Random Forest classifiers. These features are crucial in understanding customer behaviors and purchase intentions. For instance, the time spent on reading product information and engaging in administrative tasks was found to significantly influence customers’ purchase intentions.

We allocated a separate portion of our dataset for validation and reported several performance metrics, such as recall. In the context of our problem, a high recall would signify that our model successfully identified a large portion of actual positives, a crucial element in predicting customer purchasing intentions.

Cross-validation served as a robust measure to ensure the generalizability and reliability of our models. By partitioning our dataset into several subsets, we trained and tested our models multiple times, each with a different combination of subsets. This strategy allowed us to evaluate the model’s performance across different segments of the data, thus mitigating overfitting and enhancing the model’s predictive power on unseen data.

Simultaneously, parameter tuning was conducted to optimize the performance of our machine learning models. For instance, in the Random Forest classifier, key parameters such as the number of trees in the forest and the maximum number of features considered at each split were fine-tuned to attain the best model performance. This process was achieved through grid search and randomized search, techniques that systematically work through multiple combinations of parameter tunes, cross-validating as it goes to determine which tune gives the best performance.

The successful implementation of cross-validation and parameter tuning techniques contributed to the superior performance of our models. However, it is important to note that despite the meticulous tuning, the assumptions, and decisions made during this process might have influenced the model’s performance. For instance, the decision to maintain outliers in the dataset could have skewed certain model parameters, affecting the overall results.

Additionally, it is worth noting that while we achieved promising results with our chosen models and parameter settings, there may be other combinations or entirely different models that could yield even better performance. This represents another avenue for future research to further improve the predictability of online shopping behaviors.

While our study provides important insights into consumer behaviors on e-commerce platforms, it is crucial to consider the real-world applicability of our findings and the associated enablers. Our machine learning models, particularly the Random Forest classifier, can be applied by e-commerce businesses to analyze their own customer behavior data and improve their marketing strategies. The features we identified as important, such as the time spent reading product information and engaging in administrative tasks, can help businesses to understand what factors most influence their customers’ purchasing decisions.

However, successful implementation of these machine learning techniques requires certain enablers. A supply of relevant and accurate data is key, as our models depend on the quality and representativeness of the data on which they are trained. Technical expertise is also necessary, both to implement the models and to interpret their results. Given the computational complexity of machine learning models, appropriate computational resources will be required. Moreover, the cost and potential return on investment are also important considerations for businesses.

In addition, we recognize the need for seamless integration of these machine learning models into the existing e-commerce systems, which may necessitate some degree of software development and system design. Finally, while our study did not find a significant impact of bounce rates on purchase intentions, we suggest that businesses also consider other factors that were not included in our dataset but could influence customer behavior, such as the visual aesthetics of the website and the level of trust users have in the platform.

While these factors may pose challenges, they also offer opportunities for collaboration between data scientists, business analysts, and decision makers in the e-commerce industry. By working together, they can leverage the power of machine learning to enhance the online shopping experience and ultimately drive business growth.

### Comparison with Existing Methods

Our study aligns with the broader trend in e-commerce research of leveraging machine learning to understand and predict consumer behavior. However, it distinguishes itself by focusing on a unique blend of variables and methodologies. This includes a comprehensive exploratory data analysis, cluster analysis, robust machine learning models, and meticulous model validation techniques, thereby providing a holistic overview of consumer behavior in online shopping.

When compared to existing methods, our approach yields several advantages. Firstly, unlike many previous studies that focused on specific web pages or simple metrics such as page views or click-through rates, our method employed a comprehensive analysis of clickstream data. This enabled us to capture nuanced patterns and trends that may be overlooked by other methods.

Secondly, our use of multiple machine-learning models, including Decision Tree and Random Forest classifiers, allowed us to accommodate the high dimensionality and temporal dependencies inherent in clickstream data. This differs from many traditional approaches which rely on a single model and may fail to capture complex relationships within the data. Our models were not only optimized for accuracy but also for interpretability, offering meaningful insights into the factors that influence online purchasing behaviors.

However, as with any method, ours is not without limitations. Our decision to retain outliers in the dataset is a notable deviation from many existing methods, and while this decision was made to capture atypical customer behaviors, it may also have introduced noise into our models. Furthermore, our models rely on the assumption that the dataset is representative of the general online shopping population, which may not always hold true.

Comparison of our results with published results from other methods reveals a promising performance of our models. For instance, while our Random Forest classifier demonstrated a high ROC_AUC score, it was also found to be computationally efficient and robust to outliers, making it a strong competitor to models used in other studies.

In conclusion, our results underscore the importance of rigorous validation and tuning processes in machine learning analyses. By implementing these practices, we were able to develop models that not only performed well on our dataset, but also demonstrated potential for generalizing to new data, thereby offering valuable insights into online shopping behaviors and intentions.

Finally, like any analysis, ours also carries certain limitations and assumptions. For instance, we assumed that our dataset was representative of the general online shopping population. Furthermore, our analysis does not account for all potential influencing factors, such as website aesthetics or user trust. These areas, unexplored in the current study, offer fertile ground for future research.

In conclusion, our findings emphasize the need for in-depth exploration into consumer behavior within online shopping ecosystems and suggest strategies for improving user experience and purchase intentions. Future research will continue to refine these concepts and methods.

## 6. Conclusions

In conclusion, this paper has investigated the factors that influence customers’ intentions to buy or not when using e-commerce websites for shopping. By analyzing clickstream data, we have extended existing theories used for explaining online shopping intentions. We found that the time spent reading information about products and the time spent on administrative tasks are important factors that influence customer intentions. Additionally, we found that the use of Support Vector Classifier performed well in predicting customer intentions, while the information about bounce rates was not as important.

Our findings suggest that e-commerce practitioners should focus on providing well-presented and relevant information about products, as well as utilizing modern IT technologies to improve the overall customer experience. Additionally, practitioners should investigate ways to increase trust in, attractiveness, and relevance of their websites.

Future research is needed in studying customer behaviors in online shopping environments, specifically in finding better ways to present information about products and administrative aspects. Our study represents a conceptualization of “how to best detect the website pages that would benefit most from improving the informational content.” Further analysis and investigations are needed to improve this concept.

In light of the results obtained, it can be inferred that the chosen classification algorithms performed well in predicting customer intentions, with logistic regression, decision tree, random forest and support vector classifier all achieving comparable accuracy on the training and test data. The exception was the decision tree, which is known to have a tendency to overfit. However, the prediction remained very good, as it was more than 90% accurate in detecting false negatives. The main goal of finding the best predictors is related to the capacity of the classifier to offer good prediction values for the ‘true’ class, because customers who are about to exit the website pages present interest.

Another measure of interest was the recall, which aims at predicting the 1s as correctly as possible. In all cases, the recall was high, indicating that the classifiers correctly predict the defaulters. Finally, the ROC-AUC was another evaluation criterion of interest, and the AUC values were found to be very good, indicating that the classifiers were performing well in predicting both defaulters and non-defaulters.

## Figures and Tables

**Table 1:** Pseudocode.

Algorithm Steps
1. Initialize dataset
2. Conduct exploratory data analysis
2.1. Use descriptive statistics, box plots, pair plots, and clustering techniques
3. Preprocess data
3.1. Scale numerical variables with StandardScaler
4. Build Machine Learning models
4.1. For each model (Logistic Regression, Decision Tree, Random Forest, Support Vector Machines)
4.1.1. Apply GridSearchCV for parameter tuning
4.1.2. Train model on training data
4.1.3. Evaluate model on validation data
5. Determine the most significant predictors
6. Validate research hypothesis

**Table 2:** Descriptive statistics of variables.

Variable	Count	Mean	Std	Min	Max	25%	50%	75%
Administrative	12,330	2.32	3.32	0	27.00	0	1	4
Administrative_Duration	12,330	80.82	176.78	0	3398.75	0	7.5	93.26
Informational	12,330	0.50	1.27	0	24.00	0	0	0
Informational_Duration	12,330	34.47	140.75	0	2549.38	0	0	0
ProductRelated	12,330	31.73	44.48	0	705.00	7	18	38
ProductRelated_Duration	12,330	1194.75	1913.67	0	63,973.52	184.14	598.94	1464.16
BounceRates	12,330	0.02	0.05	0	0.20	0	0.00	0.02
ExitRates	12,330	0.04	0.05	0	0.20	0.01	0.03	0.05
PageValues	12,330	5.89	18.57	0	361.76	0	0	0
SpecialDay	12,330	0.06	0.20	0	1.00	0	0	0
OperatingSystems	12,330	2.12	0.91	1	8.00	2	2	3
Browser	12,330	2.36	1.72	1	13.00	2	2	2
Region	12,330	3.15	2.40	1	9.00	1	3	4
TrafficType	12,330	4.07	4.03	1	20.00	2	2	4
		unique	Top	freq				
Month	12,330	10	May	3364				
VisitorType	12,330	3	Returning_Visitor	10,551				
Weekend	12,330	2	False	9462				
Revenue (target variable)	12,330	2	False	10,422				

**Table 3:** Mean values for each variable per each cluster.

Cluster	0	1	2	3	4	5	6	7	8
Administrative	0.03	1.75	2.54	2	3.94	2.85	8.25	1.68	1.91
Administrative_Duration	0.84	56.02	74.26	66.5	187.23	99.59	343.67	51.53	57.97
Informational	0.01	0.3	0.39	0.38	0.41	0.51	3.44	0.29	0.28
Informational_Duration	0	19.18	14.87	18.77	24.45	26.75	338.8	12.3	10.48
ProductRelated	2.23	18.79	28.77	25.38	30.48	31.34	130.56	24.65	33.64
ProductRelated_Duration	32.29	749.76	980.06	942.18	1018.52	1219.5	5334.41	879.85	1247.84
BounceRates	0.19 *	0.01 *	0.01 *	0.01 *	0.01 *	0.00 *	0.01 *	0.01 *	0.01 *
ExitRates	0.19	0.03	0.03	0.03	0.03	0.02	0.02	0.04	0.03
PageValues	0	1.44	3.33	2.48	5.5	50.46	7.56	1.73	1.79
SpecialDay	0.07	0	0	0	0	0.01	0.02	0.24	0
OperatingSystems	2.18 *	2.10 *	2.09 *	2.23 *	2.04 *	2.13 *	2.11 *	2.12 *	2.10 *
Browser	2.32 *	2.29 *	2.45 *	2.54 *	2.23 *	2.71 *	2.17 *	2.37 *	2.21 *
Region	3.11 *	3.04 *	3.42 *	3.38 *	3.15 *	3.28 *	2.76 *	3.09 *	3.11 *
TrafficType	4.93 *	3.16 *	3.51 *	3.94 *	4.24	4.20 *	3.41 *	4.44 *	4.39 *
Weekend	0.17 *	0.25 *	0.21 *	0.22 *	0.27 *	0.25 *	0.27 *	0.21 *	0.26 *
Revenue	0	0.05	0.13	0.08	0.18	0.95	0.33	0.02	0.13

The values denoted with an asterisk (*) indicate that they do not show significant differences among the clusters.

**Table 4:** AUC_ROC and standard deviation of AUC_ROC computed for StratifiedKFold validation.

cv = 10	Logistic Regression	Decision Tree	Random Forest	Support Vector Classifier
AUC_ROC	0.8552	0.8738	0.9777	0.94
Standard deviation	0.052	0.064	0.016	0.044

**Table 5:** Best parameters and best accuracy after performing GridSearchCV.

	Logistic Regression	Decision Tree	RandomForest	Support Vector Classifier
ROC_AUC	0.9305	0.9343	0.954	0.9375
Best parameters	‘penalty’: ‘l2’, ‘solver’: ‘saga’	‘max_depth ‘: 9	‘max_depth’: 5, ‘max_features’: 12}	C’: 1, ‘gamma’: 1, ‘kernel’: ‘rbf’

**Table 6:** Metrics of ML classifiers on test dataset.

	Logistic Regression	Decision Tree	Random Forest	Support Vector Classifier
Accuracy on the training data	0.8745	0.9138	0.8922	0.9115
Accuracy on the test data	0.8823	0.8991	0.8973	0.9021
Recall	0.87	0.9061	0.901	0.9045
ROC-AUC	0.8822	0.8981	0.8973	0.9021
F1-score (false value)	0.88	0.9	0.9	0.9
F1-score (true value)	0.88	0.9	0.9	0.9

## Data Availability

Publicly available datasets were analyzed in this study. This data can be found here: https://archive.ics.uci.edu/ml/datasets/Online+Shoppers+Purchasing+Intention+Dataset (accessed on 1 March 2023).
